# NEIGHBORHOOD IMPACTS ON KOREAN OLDER ADULTS’ DEPRESSION

**DOI:** 10.1093/geroni/igac059.2425

**Published:** 2022-12-20

**Authors:** Seon Kim

**Affiliations:** Virginia commonwealth university, Richmond, Virginia, United States

## Abstract

Older adults are more influenced by their neighborhood than young adults, however neighborhood impacts on older adults are little known. In response to the increasing depression of older adults in Korea, this study aims to identify the effect of perceived and objective neighborhood characteristics in depression and find differences between rural and urban areas. This study uses the Korean Elderly Survey conducted in 2020 on 10,097 older adults aged 65 and over residing in 17 states, and Korean administration data by connecting them. Multilevel modeling results show that after controlling individual characteristics, depression decreases when older adults perceive their housing condition (b=-.64, p<.001), interaction with neighbors (b=-.29, p<.001), overall neighborhood environment (b=-.26, p<.001) positively. Although objective neighborhood characteristics do not significantly reduce depression in older adults living in urban area, for older adults living in rural areas, the number of social worker (b=- .42, p <.001), the number of senior center (b=-6.70, p<.001), and home care services (b=-49.5, p <.001) are negatively associated with depression in older adults. This study discusses that neighborhood characteristics may play an important role in older adults’ depression, and that their effects may differ between rural and urban areas. This study encourages policy makers to consider neighborhood characteristics to improve the mental health of Korean older adults.

